# Neurogenesis After Stroke: A Therapeutic Perspective

**DOI:** 10.1007/s12975-020-00841-w

**Published:** 2020-08-29

**Authors:** Abir A. Rahman, Narayanappa Amruta, Emmanuel Pinteaux, Gregory J. Bix

**Affiliations:** 1 Clinical Neuroscience Research Center, Department of Neurosurgery, Tulane University School of Medicine, Room 1349, 131 S. Robertson, Ste 1300, New Orleans, LA 70112 USA; 2 Faculty of Biology, Medicine and Health, University of Manchester, A.V. Hill Building, Oxford Road, Manchester, M13 9PT UK; 3 Tulane Brain Institute, Tulane University, New Orleans, LA 70112 USA

**Keywords:** Stroke, Neurogenesis, Neuroinflammation, Cytokines, Stroke therapy

## Abstract

Stroke is a major cause of death and disability worldwide. Yet therapeutic strategies available to treat stroke are very limited. There is an urgent need to develop novel therapeutics that can effectively facilitate functional recovery. The injury that results from stroke is known to induce neurogenesis in penumbra of the infarct region. There is considerable interest in harnessing this response for therapeutic purposes. This review summarizes what is currently known about stroke-induced neurogenesis and the factors that have been identified to regulate it. Additionally, some key studies in this field have been highlighted and their implications on future of stroke therapy have been discussed. There is a complex interplay between neuroinflammation and neurogenesis that dictates stroke outcome and possibly recovery. This highlights the need for a better understanding of the neuroinflammatory process and how it affects neurogenesis, as well as the need to identify new mechanisms and potential modulators. Neuroinflammatory processes and their impact on post-stroke repair have therefore also been discussed.

## Introduction

Stroke is a debilitating disease condition defined as either an interruption of blood supply to the brain due to a clot or embolism, or the rupture of a blood vessel in the brain, which then leads to neurological impairments [1]. It remains the 3rd leading cause of death worldwide, with nearly 15 million people being affected every year [2], while in the USA, it is the number 5 killer, killing nearly 140,000 people every year (https://www.cdc.gov/stroke/facts.htm). Currently, the treatment for ischemic stroke is to administer a thrombolytic agent such as tissue Plasminogen Activator (tPA) or to perform a surgical thrombectomy procedure to mechanically remove the blood clot (thrombus) [3]. However, the optimal time window for these treatments is very small and survivors often exhibit a high degree of morbidity, as well as limited functional recovery [4]. New modes of therapy are therefore urgently needed, especially ones that can be administered after longer periods following stroke onset, that can lead to better functional recovery and reduced morbidity. To this end, post-stroke brain repair processes are of particular research interest. Here, in this review, we discuss stroke-induced neurogenesis as a potential target for therapeutic intervention, as it represents a major repair mechanism that by itself falls short in achieving full recovery in surviving patients, and presumably could be modulated to achieve better outcomes. A detailed understanding of this phenomenon is needed to guide future research and the development of effective intervention strategies.

## Neurogenesis in the Post-stroke Brain

**Fig. 1:**
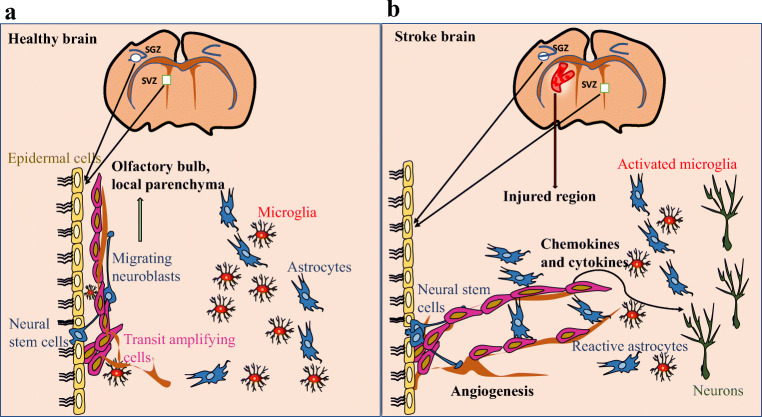
Schematic representation of adult neurogenesis in rodents. **a** Healthy brains: Neural stem cells proliferate from the SVZ and SGZ form neuroblasts that migrate to the olfactory bulb and local parenchyma. **b** Stroke brains: There is pronounced loss of striatal and cortical neurons, giving rise to increased proliferation of progenitors. The neuroblasts formed before and after the stroke migrate to the site of injury, influenced by chemokines and cytokines secreted by resident and activated microglia, reactive astrocytes, etc. The neuroblasts then differentiate into newborn neurons coupled with angiogenesis at the site of injury, giving rise to mature neurons

Neurogenesis, or the birth of new neurons, is known to be induced in response to ischemic stroke, in the infarct and surrounding areas. Neural stem cells originating from the sub-ventricular zone (SVZ) and the sub-granular zone of the dentate gyrus are considered to give rise to these new neurons [5–7]. This is thought to be a key process in post-stroke recovery and repair of the damaged brain region [8–10]. In general, this involves the migration of neural stem cells to the infarct and peri-infarct region, followed by their differentiation into functional neurons [11–13]. This process is schematically depicted in Fig. 1, based on information derived from research in rodents and other model systems. An important factor to consider in post-stroke functional recovery is the ultimate survival of these newborn neurons. Several studies have reported their reduced survivability possibly due to their microenvironment lacking trophic factors as well as chronic inflammatory responses [14–16]. Acute neuroinflammation, however, has been reported to promote neurogenesis and may be promoting neuronal survival as well [17, 18]. In addition, age remains a prominent factor affecting neurogenesis. The rate of neurogenesis steadily declines with rising age, with stroke increasing the sharpness of that decline [8, 19, 20]. Notably, Darsalia and collaborators [8] reported that striatal neurogenesis after stroke is similar in young and aged mice, while hippocampal neurogenesis is impaired in aged animals compared with the young animals. This raises the possibility of differential region-specific regulation mechanisms and multiple modulatory opportunities if the mechanisms could be harnessed.

## Factors Governing Neurogenesis

The process of neurogenesis can generally be categorized into three stages: (1) neural stem cell proliferation, (2) migration of neuroblasts and immature neurons, and (3) differentiation into mature neurons and neurite extension, finally leading to synaptogenesis and stabilization of the synapses. There are a number of molecules that affect one or more of these stages, and they differ between embryonic neurogenesis and adult neurogenesis [21–23]. We will focus on some that have been identified as important for stroke-induced neurogenesis.

Ruan and collaborator in a recent review [24] mention Fibroblast growth factor-2 (FGF-2) [25, 26], Insulin-like Growth Factor-1 (IGF-1) [27, 28], Brain-Derived Neurotrophic Factor (BDNF) [29–31], and Vascular Endothelial Growth Factor (VEGF) [32] as factors that directly affect neural stem cell proliferation, while identifying Stromal-derived factor (SDF-1), Monocyte Chemoattractant Protein (MCP-1), and Matrix metalloproteinases (MMP) 2, 3, and 9 as factors influencing neuroblast migration [33–36]. SDF-1 and MCP-1 are both chemokines that form part of the inflammatory response to the ischemic injury [37, 38], whereas MMPs are matrix metalloproteinases involved in remodeling of the extracellular matrix [35]. Remodeling of the matrix often occurs to allow reparative processes like angiogenesis to take place [39]. In this case, however, the remodeling may be taking place in part to allow the migrating neuroblasts to pass through. In addition, proteolysis of matrix proteins such as perlecan has been implicated in promoting neurogenesis in post-stroke brains [40]. Its c-terminal domain V (DV) is thought to be the active component leading to neurogenesis as well as angiogenesis at the infarct and peri-infarct area [41, 42]. Using neurospheres and fetal cortical neurons in vitro, Trout and colleagues [42] showed that perlecan DV also promoted differentiation into mature neurons as well as neurite extension. In this way, the MMPs may be serving a dual function.

## Neuroinflammation and Neurogenesis

For the most part, the dogma is that neuroinflammation and neurogenesis are inversely related. Factors like Sirtuin 7, Glucagon-Like Peptide-1, and Nei like DNA Glycosylase 1 enhance neurogenesis by suppressing neuroinflammation [43–45]. Inhibition of Glycogen Synthase Kinase-3 (GSK-3) has also been shown to increase neurogenesis, while reducing neuroinflammation, implicating Wnt signaling in the process [46–48]. However, depending on the timing, duration, and the profile of cytokines and chemokines released, neurogenesis could be positively impacted [17, 18, 49–51].

An example of how the duration of inflammation can be an important factor is seen by the effect of interleukin-6 (IL-6) on neurogenesis. Short-term treatment of neural stem cells with a hyperactive fusion IL-6 protein induces neurogenesis in vitro [49], while chronic astrocytic IL-6 transgene expression led to reduced neurogenesis in the dentate gyrus of the transgenic mice [20]. Similarly, interleukin 1α (IL-1α) and interleukin-1β (IL-1β) follow an out of phase expression pattern in response to ischemia, where IL-1α expression occurs early following ischemia whereas IL-1β is expressed much later and coincides with reduced IL-1α production, indicating that the two cytokines likely have different roles during the post-stroke neuroinflammatory response [52, 53]. Indeed, IL-1α is reported to be neurogenic [17] and gliogenic [54], while IL-1β is primarily thought to induce neural death and clearance of dead cells and debris [55–57]. One of the ways interleukin-1 (IL-1) potentially regulates neurogenesis is by driving the expression of pentraxin 3 (PTX-3). This protein is a known biomarker for cerebrovascular diseases and plays a key role in maintaining blood-brain barrier (BBB) integrity. While one study identified IL-1 as a driving regulator of PTX-3 [58], a second follow-up study reported the neurogenic ability of PTX-3, inducing IL-1β-dependent proliferation in neurospheres [59]. In the latter study, PTX3 knockout mice also exhibited reduced proliferating stem cell population in the dentate gyrus after MCAO, further supporting the idea that PTX-3 induces neurogenesis.

Among the various cells involved in mediating inflammation in the brain, microglia are of particular interest as a cell type that can infer both neuroprotection and neuronal death. How they affect neurogenesis has been a major focus of many studies. Microglia have been reported to produce trophic factors to guide and support neural stem cell migration and differentiation, but also, on other occasions, produce cytokines that hamper cell survival [60–64]. When microglia in the brain were nearly completely depleted by administration of the Colony-stimulating factor 1 receptor (CSF1R) antagonist PLX3397, the resulting mice exhibited approximately 60% increase in infarct size after MCAO [65], indicating the largely neuroprotective role played by microglia. In a quiescent and ramified state, microglia tend to secrete trophic factors that support surrounding neurons, whereas in a more activated (ameboid) state, they tend to eliminate neurons. The CX3C Receptor 1, expressed by microglia, has been shown to play a key role in the modulation of these characteristics. Its inhibition leads to reduced hippocampal neurogenesis leaving the olfactory bulb unchanged [66, 67].

In addition to microglia, astrocytes have also been shown to influence neurogenesis, particularly reactive astrocytes [68, 69]. Traditionally, they were thought to contribute more to neuronal apoptosis than neuronal survival in the ischemic brain [70]. However, recent studies have informed more about their robust neurogenic properties. In addition to secreting growth and other factors to strengthen synapses, some astrocytes act as neuronal precursors [71–73]. These cells can not only differentiate into mature neurons but are also able to divide asymmetrically to generate a neuron and another precursor [73]. There are ongoing efforts to exploit these properties clinically, especially in conjunction with stem cell–based approaches [74]. Faiz and colleagues (2015) have identified Ascl1 as a gene that can induce the transdifferentiation of astrocytes into neurons [75]. Similarly, Zhang et al. (2018) have implicated IL-17 expression and release from astrocytes in promoting neurogenesis via the NF-κB pathway [76]. Another study reported that the secretion of β-arrestin from astrocytes promoted neurogenesis, while knockout animals displayed reduced proliferation of neural precursor cells [77]. Disrupted in Schizophrenia 1 (DISC-1) is yet another gene expressed by astrocytes known to influence neurogenesis, where a dominant negative mutation is known to cause reduced neurogenesis [78]. Astrocytes, therefore, present an attractive target for stroke therapy, especially when combined with stem cell–based therapeutic approaches and other neurogenesis promoting treatments. If the developmental ability of astrocytes to neurons can be harnessed and modulated, this presents a somewhat renewable pool of neural precursors, which is additional to neuroblasts from the SVZ and the DG. Furthermore, astrocyte-derived growth and support factors can also be clinically targeted to ensure better survival of the post-stroke newborn neurons.

## Targeting Neurogenesis in Experimental Stroke

Regarding modulating post-stroke neurogenesis to improve stroke outcome, there are a number of considerations. Selective ablation of post-stroke neurogenesis has been reported to have deleterious effects in stroke recovery [79–81], indicating the potential for manipulating neurogenesis to alter stroke outcome. This is further reinforced by studies showing enhancement of neurogenesis positively affecting stroke outcome [82–85]. In doing these studies, the timing of intervention becomes a key consideration because neurogenesis is a part of the delayed repair process and any intervention targeting an aspect of the neurogenic process needs to be in synchrony for maximum efficacy [86–88]. In addition, it is important to consider the interplay with other repair processes such as angiogenesis [24]. Some recent studies examining post-stroke neurogenesis are listed in Table 1.

**Table 1:** Recent studies investigating post-stroke neurogenesis for therapy

Title	Means of neurogenesis modulation	Model/mice used for study	Study outcome/observations	Citation
Aryl Hydrocarbon Receptor Modulates Stroke-Induced Astrogliosis and Neurogenesis in the Adult Mouse Brain	Use of an AHR conditional knockout mouse and the administration of an AHR antagonist called TMF (5 mg/kg/day in 2% DMSO, for 2 days)	Permanent MCAO model: C57/Bl6 mice – Wild-type mice and Nestin-CreERT2/AHR-Flox mice	Both the conditional knockout and treatment with an AHR antagonist led to improved stroke outcome and increased neurogenesis, whereas activation of the AHR led to reduced neurogenesis and increased astrogliosis	Chen WC, Chang LH, Huang SS, Huang YJ, Chih CL, Kuo HC, Lee YH, Lee IH. Aryl hydrocarbon receptor modulates stroke-induced astrogliosis and neurogenesis in the adult mouse brain. *J Neuroinflammation*. 2019 Oct 12;16(1):187. doi: 10.1186/s12974-019-1572-7. PMID: 31606043; PMCID: PMC6790016. [142]
Ergostatrien-7,9(11),22-trien-3β-ol From Antrodia Camphorata Ameliorates Ischemic Stroke Brain Injury via Downregulation of p65NF-κ-B and Caspase 3, and Activation of Akt/GSK3/catenin-associated Neurogenesis	Treatment with crude extract from *Antrodia camphorata* at 0.3 g/kg or 0.6 g/kg and its active ingredient ergostatrien-7,9(11),22-trien-3β-ol at 60 mg/kg or 120 mg/kg, all given orally	Transient focal MCAO model: Male ICR mice	Both compounds were shown to reduce ischemic brain injury by decreasing p65NF-κB and caspase 3 expression, and they promote neurogenesis (DCX) and neuroprotection (Bcl2) by activating the PI3k/Akt-associated GSK3 inhibition and β-catenin activation	Wang YH, Chern CM, Liou KT, Kuo YH, Shen YC. Ergostatrien-7,9(11),22-trien-3β-ol from Antrodia camphorata ameliorates ischemic stroke brain injury via downregulation of p65NF-κ-B and caspase 3, and activation of Akt/GSK3/catenin-associated neurogenesis. *Food Funct*. 2019 Aug 1;10(8):4725–4738. doi: 10.1039/c9fo00908f. Epub 2019 Jul 15. PMID: 31304955. [144]
Abolition of aberrant neurogenesis ameliorates cognitive impairment after stroke in mice	Exercise, administration of pharmacological agents temozolomide (4 rounds of treatment at 1-week intervals, with each round consisting of 1 injection of TMZ (0.9% in saline containing 10% DMSO, 25 mg/kg, i.p.) per day for 4 consecutive days) and mementine ((0.9% saline, 25 mg/kg, i.p.) once per week over 4 weeks), and conditional genetic deletion of the diphtheria toxin fragment A (DTA) cassette gene, causing apoptosis in NSCs	Permanent MCAO model: C57/Bl6 mice – Wild-type mice and Nestin-CreERT2/NSE-Stop-DTA transgenic mice	Stroke induces hippocampal (SGZ) neurogenesis which seems to cause memory impairments. Enhancement of neurogenesis with mementine and exercise on running wheel led to poorer cognitive performance after stroke. In contrast, reduction of neurogenesis using temozolomide and a transgenic apoptotic induction led to better memory retrieval.	Cuartero MI, de la Parra J, Pérez-Ruiz A, Bravo-Ferrer I, Durán-Laforet V, García-Culebras A, García-Segura JM, Dhaliwal J, Frankland PW, Lizasoain I, Moro MÁ. Abolition of aberrant neurogenesis ameliorates cognitive impairment after stroke in mice. *J Clin Invest*. 2019 Apr 1;129(4):1536–1550. doi: 10.1172/JCI120412. Epub 2019 Feb 25. PMID: 30676325; PMCID: PMC6436875. [143]
MEPO Promotes Neurogenesis and Angiogenesis but Suppresses Gliogenesis in Mice With Acute Ischemic Stroke	Erythropoeitin (EPO) and mutant erythropoeitin (MEPO) administration—5000 U/kg	Transient middle cerebral artery occlusion: Male C57BL/6 mice	EPO and MEPO both increased the regeneration of neurons and blood vessels in peri-infarct region, and suppressed gliogenesis, leading to improved neurological function and neuronal survival rate	Zhang SJ, Wang RL, Zhao HP, Tao Z, Li JC, Ju F, Han ZP, Ma QF, Liu P, Ma SB, Cao GD, Luo YM. MEPO promotes neurogenesis and angiogenesis but suppresses gliogenesis in mice with acute ischemic stroke. *Eur J Pharmacol*. 2019 Apr 15;849:1–10. doi: 10.1016/j.ejphar.2019.01.066. Epub 2019 Feb 2. PMID: 30716313. [95]
Docosanoids Promote Neurogenesis and Angiogenesis, Blood-Brain Barrier Integrity, Penumbra Protection, and Neurobehavioral Recovery After Experimental Ischemic Stroke	Docosahexaenoic acid (DHA) at 5 mg/kg given IV and neuroprotectin D1 (NPD1) at 5 μg/rat treatment, given ICV	Transient middle cerebral artery occlusion: Male Sprague-Dawley Rats	DHA was found to increase neurogenesis in the cortical and subcortical peri-infarct regions. It also led to an activation of NPD1 synthesis in the same regions, which in turn, was found to enhance neurogenesis and axonal regeneration, while reducing BBB permeability, when given exogenously.	Belayev L, Hong SH, Menghani H, Marcell SJ, Obenaus A, Freitas RS, Khoutorova L, Balaszczuk V, Jun B, Oriá RB, Bazan NG. Docosanoids Promote Neurogenesis and Angiogenesis, Blood-Brain Barrier Integrity, Penumbra Protection, and Neurobehavioral Recovery After Experimental Ischemic Stroke. *Mol Neurobiol*. 2018 Aug;55(8):7090–7106. doi: 10.1007/s12035-018-1136-3. Epub 2018 Jun 1. PMID: 29858774; PMCID: PMC6054805. [92]
Photobiomodulation Therapy Promotes Neurogenesis by Improving Post-Stroke Local Microenvironment and Stimulating Neuroprogenitor Cells	Cold white light (808 nm) treatment at the scalp applied for 2 min daily	Photothrombotic stroke model: Male Sprague-Dawley rats	PBM was able to reduce infarct volume and behavioral deficits after stroke, while increasing neurogenesis and synaptogenesis. Reactive gliosis, release of both pro- and anti-inflammatory cytokines, and cytochrome c oxidase were shown to underlie the beneficial effects of PBM.	Yang L, Tucker D, Dong Y, Wu C, Lu Y, Li Y, Zhang J, Liu TC, Zhang Q. Photobiomodulation therapy promotes neurogenesis by improving post-stroke local microenvironment and stimulating neuroprogenitor cells. *Exp Neurol*. 2018 Jan;299(Pt A):86–96. doi: 10.1016/j.expneurol.2017.10.013. Epub 2017 Oct 19. PMID: 29056360; PMCID: PMC5723531. [146]
Pyruvate Kinase M2 Increases Angiogenesis, Neurogenesis, and Functional Recovery Mediated by Upregulation of STAT3 and Focal Adhesion Kinase Activities After Ischemic Stroke in Adult Mice	Recombinant Pyruvate Kinase M2 administration (160 ng/kg), delivered intranasally	Permanent MCAO model: C57/Bl6 mice - Wild type	rPKM2 was neuroprotective after stroke. It also activated STAT3 and promoted angiogenesis via this mechanism. It was also shown to promote NPC migration mediated by Focal Adhesion Kinase expression, in vitro as well as in vivo. Neuronal differentiation and local cerebral blood flow were also shown to be promoted in the ischemic brain by rPKM2.	Chen D, Wei L, Liu ZR, Yang JJ, Gu X, Wei ZZ, Liu LP, Yu SP. Pyruvate Kinase M2 Increases Angiogenesis, Neurogenesis, and Functional Recovery Mediated by Upregulation of STAT3 and Focal Adhesion Kinase Activities After Ischemic Stroke in Adult Mice. *Neurotherapeutics*. 2018 Jul;15(3):770–784. doi: 10.1007/s13311-018-0635-2. Erratum in: *Neurotherapeutics*. 2018 Jun 26;: PMID: 29869055; PMCID: PMC6095793. [85]
Enriched Housing Promotes Post-Stroke Neurogenesis Through Calpain 1-STAT3/HIF-1α/VEGF Signaling	An enriched housing environment that included social interactions, voluntary and varied physical activity, and introduction of novel objects to provide physical, social, and cognitive stimulation. Additional treatments were Calpain-1 inhibitor PD151746 (0.2 mg/kg dissolved in 1% DMSO), JAK/STAT3 pathway inhibitor AG490 (5 mg/kg), HIF1α inhibitor 2-methoxyestradiol (5 mg/kg), anti-VEGF neutralizing antibody (1 μg/μl), and glycyrrhizin (10 mg/mouse), all given ICV.	Transient middle cerebral artery occlusion: Male C57BL/6 mice	Enriched housing conditions were found to facilitate post-stroke functional recovery, and this was mediated by Calpain-1, which modulated STAT3, HIF1α, VEGF, and HMGB1 levels, to result in increased neurogenesis.	Wu X, Liu S, Hu Z, Zhu G, Zheng G, Wang G. Enriched housing promotes post-stroke neurogenesis through calpain 1-STAT3/HIF-1α/VEGF signaling. *Brain Res Bull*. 2018 May;139:133–143. doi: 10.1016/j.brainresbull.2018.02.018. Epub 2018 Mar 22. PMID: 29477834. [145]
Cannabinoid Type-2 Receptor Drives Neurogenesis and Improves Functional Outcome After Stroke	Administration of cannabinoid type 2 receptor (CB2R) agonist JWH133 (1.5 mg/kg) and the antagonist SR144528 (5 mg/kg), and CB2R genetic deletion.	Permanent MCAO model: C57/Bl6 mice - Wild type and CB2R KO transgenic	Pharmacological inhibition and genetic deletion of CB2R both led to reduced neuronal migration and neurogenesis after stroke. Treatment with the agonist did not have an effect and the antagonist had no effect on neurogenesis in vitro.	Bravo-Ferrer I, Cuartero MI, Zarruk JG, Pradillo JM, Hurtado O, Romera VG, Díaz-Alonso J, García-Segura JM, Guzmán M, Lizasoain I, Galve-Roperh I, Moro MA. Cannabinoid Type-2 Receptor Drives Neurogenesis and Improves Functional Outcome After Stroke. *Stroke*. 2017 Jan;48(1):204–212. doi: 10.1161/STROKEAHA.116.014793. Epub 2016 Nov 29. PMID: 27899748. [10]
MicroRNA Cluster miR-17-92 Cluster in Exosomes Enhance Neuroplasticity and Functional Recovery After Stroke in Rats	Treatment with exosomes containing MicroRNA 17–92 or control MSC-derived exosomes (100 μg total exosome protein, per rats), administered IV	Transient MCAO model: Male Wistar rats.	Treatment with exosomes containing microRNA 17–92 resulted in improved neurological function, enhanced oligodendrogenesis, neurogenesis, and neurite remodeling, and increased neuronal dendrite plasticity in the ischemic boundary zone	Xin H, Katakowski M, Wang F, Qian JY, Liu XS, Ali MM, Buller B, Zhang ZG, Chopp M. MicroRNA cluster miR-17-92 Cluster in Exosomes Enhance Neuroplasticity and Functional Recovery After Stroke in Rats. *Stroke*. 2017 Mar;48(3):747–753. doi: 10.1161/STROKEAHA.116.015204. Erratum in: Stroke. 2017 May;48(5):e137. PMID: 28232590; PMCID: PMC5330787. [147]
Post-stroke Constraint-induced Movement Therapy Increases Functional Recovery, Angiogenesis, and Neurogenesis With Enhanced Expression of HIF-1α and VEGF	Constraint-induced movement therapy using a plaster cast placed on the unimpaired limb after MCAO	Transient MCAO model: Male Sprague-Dawley rats.	Constraint-induced movement therapy resulted in increased lengths of microvessels and showed evidence of enhanced neurogenesis, concomitant with an increase in HIF-1α and VEGF expression and FIH-1 expression	Li C, Zhang B, Zhu Y, Li Y, Liu P, Gao B, Tian S, Du L, Bai Y. Post-stroke Constraint-induced Movement Therapy Increases Functional Recovery, Angiogenesis, and Neurogenesis with Enhanced Expression of HIF-1α and VEGF. *Curr Neurovasc Res*. 2017;14(4):368–377. doi: 10.2174/1567202614666171128120558. PMID: 29189156. [93]

In early studies, newborn neurons were detected in gerbil brains after cerebral ischemia, 26 days after ischemic insult and persisted for 7 months [89]. More recent studies, however, showed that in mice and rats, neural stem cell proliferation in the SVZ was significantly enhanced in as early as the first 7–14 days after MCAO [11, 13, 90, 91]. Thored and colleagues reported the presence of neuroblasts from 1 week up to 16 weeks after insult, in the striatum [7]. Similarly, neuroblasts were shown to migrate to the cortex and survive for 35 weeks or more [90]. Therefore, these represent the rodent counterparts for therapeutic windows for treatments aimed at proliferation (7 days and beyond) and migration. These time windows would be different in humans and need to be investigated to determine effective treatment regimes. Lastly, most of these neurons die within 2–5 weeks [15]. It is this critical period that must be targeted if neuroprotective factors or factors promoting neuronal survival are to be administered for therapy, although it is unclear how long that treatment will have to be continued, to ensure the survival of the newborn neurons. It may be important to target the process of synaptogenesis at time periods like this since failure to make connections has been proposed as a mechanism of neuronal death [15].

The process of angiogenesis has been reported to be closely intertwined with the process of neurogenesis after stroke [24, 85, 92–95]. This process is defined as the formation of new blood vessels via the sprouting of preexisting vessels and generally occurs in response to an injury such as cerebral ischemia [96]. It is characterized by proliferation of endothelial cells that then form tube-like structures, ultimately forming the complete blood vessel [97]. Research has shown that endothelial cells secrete a number of neurotrophic factors like VEGF, Angiopoeitin-1, and SDF-1 that facilitate neurogenesis and neuronal differentiation and subsequent survival [33, 98, 99]. Moreover, angiogenic processes precede the neurogenic processes in the infarct area after ischemic insult [100–103]. Research has also shown that modulation of the factors governing neurogenesis also affects angiogenesis [32, 104–108]. These all point to the idea that modulation of neurogenesis can be achieved by modulation of angiogenesis as well as the importance of keeping angiogenic processes in mind while manipulating neurogenesis. A question can be raised about how one could go about targeting neurogenesis specifically without altering angiogenesis. One way to do so may be to delay treatments such that period of angiogenesis is surpassed and mostly neurogenesis is targeted [17], while an alternate method could involve treating with a cocktail with components that would compensate for effects on angiogenesis specifically that might occur as a concurrent effect of one or more of the other components. It might have to be a combination of the two methods or others to achieve maximum targeting efficiency.

## Drugs Targeting Neuroinflammation to Alter Neurogenesis

Minocycline is a tetracycline derivative that inhibits microglial activation and has been shown to be neuroprotective following focal cerebral ischemia [109, 110]. It has also been shown to be able to upregulate neurogenesis in multiple models [111–113]. Therefore, this remains the most promising pharmacological agent in this regard. Srivastava and collaborators [114] reported it as safe and efficacious in their clinical trial, which was further supported by the meta-analysis conducted by Malhotra and colleagues [115], of seven randomized clinical trials. It is safe to be administered for sure, but its efficacy still needs some more validation before it is widely accepted for treatment of stroke.

Another study reported that the drug Sildenafil promoted neurogenesis and was able to enhance functional recovery after perinatal/pediatric ischemia in mice [116]. While this is not exactly the same as the hypoxia occurring during ischemic stroke, it is consistent with previous findings that sildenafil promotes neurogenesis after focal cerebral ischemia [117–119]. Engels and collaborators [116] proposed that Sildenafil altered the levels of Wnt signaling pathway members β-catenin and GSK-3, via inhibition of phosphodiesterase type 5, and subsequent increase in cGMP levels. Although they did not find any direct evidence of affected neuroinflammation, GSK-3 and Wnt signaling has been implicated in the regulation of neuroinflammation in several studies [120, 121]. One GSK inhibitor called Tideglusib has been investigated in clinical trials as well, where it was deemed clinically safe but was not effective [122, 123]. Other GSK inhibitors that may be worth exploring include 6-bromoindirubin-30-oxime (BIO) [46, 48] and lithium chloride [47, 124].

Based on several in vitro and in vivo studies investigating the role of IL-1 in stroke, recent studies have considered IL-1 receptor antagonist (IL-1Ra) as an attractive new therapy. Indeed, a small phase 2 clinical trial showed that IL-1Ra is safe in stroke and may be effective [125], and the more recent SCIL-STROKE study confirmed this hypothesis that IL-1Ra may be potent neuroprotective therapy in stroke [126]. IL-1Ra may improve stroke outcome through inhibition of the inflammatory response; however, an interesting recent study found that IL-1Ra administration in rat stroke model potently promotes long-term neurogenesis and functional recovery [127]. This study suggests that, although acute inflammation is an important trigger for post-stroke neurogenesis, a more controlled neuroinflammatory response appears critical for an optimum neurogenic response after stroke. These are only a few of the drugs being tested for efficacy in stroke treatment, via neurogenesis modulation. It is widely recognized that pharmacological modulation of neurogenesis can be a valuable tool to treat stroke and is, therefore, likely to be active area of research focus for the foreseeable future.

## Potential of Stem Cell–Based Therapy and Considerations of Challenges

One major observation in ischemia-induced neurogenesis is that only a small quantity of the newborn neurons survive in the peri-infarct area [7, 15]. Therefore, to overcome this, in addition to modulating endogenous neurogenesis, stem cell therapy to treat stroke has also been investigated as a possible alternative or as a potential way to augment the endogenous stroke-induced neurogenesis [128–130].These exogenous stem cells can become the source of some much-needed trophic factors and exert paracrine reparative effects. In turn, this could lead to the microenvironment in the peri-infarct area more supportive of new neuron differentiation and integration into the circuitry.

In one study, transplanting human fetal neural stem cells into the hippocampus 24 h after surgically occluding the middle cerebral artery in mice was reported to have improved behavioral recovery and reduced infarct volume, compared with animals without the transplant [131]. They also noted improved BBB repair and lower number of activated microglia in the transplanted brains, as well as higher abundance of Brain derived neurotrophic factor (BDNF). A subsequent study by the same research group reported the transplant procedure as highly beneficial in combination with tissue plasminogen activator (t-PA) treatment, resulting in lower levels of pro-inflammatory cytokines, tumor necrosis factor (TNF-α) and IL-6, as well as MMP-9 [129]. Taken together, these validate the potential of transplanting fetal neural stem cells as a mode of therapy. Of course, the ethical challenges of obtaining and maintaining such stem cells remain a major limitation of such a process.

The alternative approach to using fetal stem cells is to use inducible pluripotent stem cells (iPSCs) or mesenchymal stem cells (MSCs). Oki et al. [132] used human iPSC-derived neuroepithelial-like stem cells that they transplanted into mice 1 week and 48 h after MCAO. They reported improved forelimb motion recovery, increased VEG-F deposition, and successful differentiation of iPSCs into neurons in the striatum.

Use of MSCs is limited by the observation that most of the systemically transplanted MSCs end up in the lungs and do not make it to the infarct area of the brain [133, 134], where they are actually intended to proliferate and repair the damage. Tobin and colleagues [135] have proposed the use of MSCs that have been activated by Interferon gamma (aMSCs). They reported that both activated and naïve MSCs induced complete behavioral recovery, reduced infarct volumes, and reduced microglial activation and levels of IL-1β, TNF-α, and IL-6 in treated animals, compared with vehicle-treated control stroke animals. However, they propose the activated MSCs are a better treatment option than naïve MSCs because of an increased yield of anti-inflammatory factors from microglia. Interestingly, they did not observe any induction of neurogenesis in the SVZ after MSC treatment.

A phase 1 clinical trial (PISCES) involving the administration of CTX0E03 human neural stem cells via stereotactic ipsilateral putamen injection reported that a dose of up to 20,000 cells is safe and well tolerated in patients [136]. The treatment also resulted in functional improvements and upon further investigation, may very well become a mainstream intervention strategy. Since the study was conducted only on 11 men, it needs to be followed up with the inclusion of female patients and a larger patient population [136].

Another phase 2 clinical trial involving the administration of bone marrow stem cells to stroke patients proved safe in patients, but ineffective in terms of treating stroke [137]. Similar results were obtained in another phase 2 clinical trial where patients were treated with bone marrow derived ALD-401 stem cells [138]. Taken together, these studies indicate that the administration of stem cells is safe. As for effectiveness, there is potential for the stem cells to promote functional recovery in more tightly controlled settings, which was a limitation of all three studies, along with the small population of patients that have been tested.

The process of preconditioning the MSCs and using the resulting media may prove even more effective in stroke treatment [128, 139]. A recent systemic review highlighted the therapeutic potential of extracellular vesicles secreted by various cells like MSCs, macrophages, and neural stem cells, identifying these vesicles as an attractive approach. However, being a recent trend, there is a significant amount of heterogeneity among the results of applications, presumably due to isolation and administration techniques, as well as cell-type of origin [140]. Further work on MSCs preconditioning with various inflammatory mediators found that IL-1α can be used as a key priming stimulus to induce MSCs to produce anti-inflammatory and neurotrophic factors [141], and a further in vivo study in mice demonstrated that conditioned medium of IL-1α-primed MSCs administered peripherally after stroke had beneficial effects on stroke outcome and functional recovery [139]. Further work investigating the efficacy of targeted delivery of IL-1α-primed MSCs in stroke is ongoing.

## Conclusion and Future Therapeutic Perspective

Stroke affects millions of people every year. With the world populations steadily rising, the global burden of stroke keeps rising proportionally. As a result, there remains a global and critical need to develop better treatment options. Stroke-induced neurogenesis presents a promising therapeutic target, since it can allow the brain to, essentially, rewire and refresh itself, and heal the damage caused by the ischemia or hemorrhage. However, harnessing neurogenesis remains a challenge because of the intricate interplay of the factors involved, especially ones involved in neuroinflammation. It is now well understood that the two processes much more deeply connected than a simple inverse relationship. Moreover, both processes are interconnected with angiogenesis and together work towards post-stroke brain repair. In order to harness them and improve functional recovery, it is imperative, now more than ever, to characterize the roles played by each immune cell, cytokine, and chemokine, as part of the post-injury microenvironment, taking into special consideration their temporal expression patterns, specific effects on angiogenesis, neurogenesis, neuroprotection, and neuron elimination. All of these need to be considered carefully to craft effective therapeutic cocktails that are to achieve maximum treatment efficiency.
